# Bilateral IgG4‐Related Dacryoadenitis

**DOI:** 10.1002/ccr3.73283

**Published:** 2026-08-03

**Authors:** Yan Song, Lin Ding, Tao Shen

**Affiliations:** ^1^ People's Hospital of Xinjiang Uygur Autonomous Region Urumqi Xinjiang China; ^2^ State Key Laboratory of Ophthalmology, Zhongshan Ophthalmic Center, Sun Yat‐sen University, Guangdong Provincial Key Laboratory of Ophthalmology and Visual Science, Guangdong Provincial Clinical Research Center for Ocular Diseases Guangzhou China

**Keywords:** dacryoadenitis, exophthalmos, IgG4‐related disease, lacrimal gland

## Abstract

Chronic dacryoadenitis is an uncommon inflammation of the lacrimal gland that may have various etiologies with similar presentations. IgG4‐related ophthalmic disease most often manifests as painless lacrimal gland enlargement, so the clinicians should consider IgG4‐related disease as a diagnostic possibility for dacryoadenitis.

## 1 Case Presentation

**FIGURE 1:**
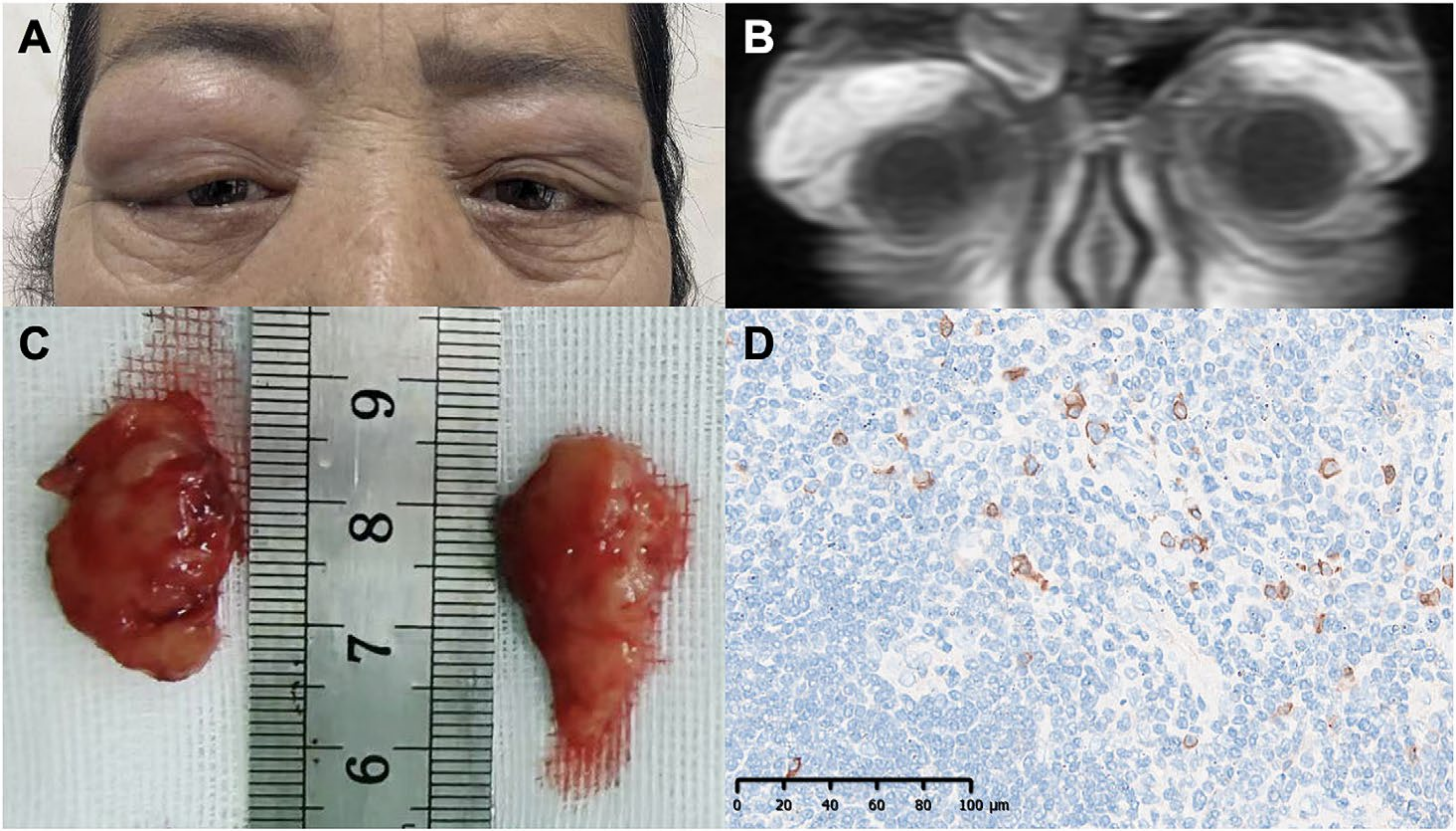
(A) Swelling of bilateral upper eyelids. (B) Coronal magnetic resonance imaging of the orbits. (C) Resected lacrimal gland masses. (D) Immunohistochemistry demonstrated IgG4‐related dacryoadenitis. Note that more than 70 plasma cells were stained with anti‐IgG4 per high‐power field (a magnification of ×200).

A 56‐year‐old woman presented with a 3‐year history of progressive, painless swelling of both upper eyelids (Figure 1A). The swellings were firm without tenderness. Visual acuity and ocular motility were normal in both eyes. Coronal magnetic resonance imaging of the orbits (Figure 1B) demonstrated swelling of the bilateral lacrimal glands without definite thickening or enhancement of the bilateral infraorbital nerves. She had no remarkable past ocular or systemic medical history. The serum nuclear antigen antibodies, rheumatoid factors, and complement factors (C3 and C4) were all within normal limits. The thyroid function was also normal. However, the serum IgG4 level was 450 mg/dL (reference range, 8–140). A full‐body PET‐CT was not performed. The patient showed no obvious response to short‐term oral prednisone (0.5 mg/kg/d, for 2 weeks). Due to the atypical clinical manifestations, bilateral lacrimal gland masses were resected to clarify the pathological diagnosis and relieve local compression (Figure 1C), and the immunohistochemistry showed more than 70 IgG4‐positive plasma cells per high‐power field (Figure 1D). Bilateral exophthalmos in the patient was significantly improved after the surgery. Prednisone was administered postoperatively (initially 30 mg/day, gradually reduced, for a total of 6 months). After 12 months of follow‐up, there was no recurrence, and serum IgG4 returned to the normal range.

Chronic dacryoadenitis is an uncommon inflammation of the lacrimal gland that may have various etiologies with similar presentations. The differential diagnosis includes nonspecific conditions and specific dacryoadenitis such as sarcoidosis, Graves' ophthalmopathy, and Sjögren's syndrome. In the present case, the decision to test the serum IgG4 level and the immunohistochemistry of the resected lacrimal gland masses proved to be critical for the definite diagnosis.

IgG4‐related disease, which was first coined in 2003, is a systemic fibroinflammatory condition characterized by IgG4‐positive plasma cell infiltration [1]. IgG4‐related disease was first described as autoimmune pancreatitis with extensive organ involvement, and a diverse range of extra‐pancreatic manifestations has been reported, including sclerosing cholangitis, pulmonary nodules, pericarditis, interstitial nephritis, renal pseudotumor, thyroiditis, retroperitoneal fibrosis, and ocular manifestations [2]. IgG4‐related ophthalmic disease most often manifests as painless lacrimal gland enlargement, but may involve other tissues such as extraocular muscles, orbital fat, and nerves [3]. According to the diagnostic criteria for IgG4‐related diseases, the following three conditions must be met: First, diffuse or focal swelling or masses occur in a single or multiple organs; second, the serum IgG4 concentration is ≥ 1350 mg/L; third, histopathological examination shows obvious lymphocyte and plasma cell infiltration accompanied by fibrosis, and IgG4‐positive plasma cells infiltration can be observed, with the ratio of IgG4‐positive cells to IgG‐positive cells being greater than 40%, and the number of IgG4‐positive plasma cells per high‐power field is more than 10. If all three conditions are met, a diagnosis can be made; if only the first and third conditions are met, it is considered highly suspected; if only the first and second conditions are met, it is a possible diagnosis.

We recommend that clinicians consider IgG4‐related disease as a diagnostic possibility for glandular pathology, especially for dacryoadenitis. The serum IgG4 level elevation may provide a useful diagnostic clue, but the infiltration of IgG4‐positive plasma cells remains the gold‐standard for diagnosis.

## Author Contributions

**Yan Song:** conceptualization, data curation, formal analysis, investigation, writing – original draft. **Lin Ding:** conceptualization, investigation. **Tao Shen:** conceptualization, formal analysis, investigation, writing – review and editing.

## Funding

This study was supported by Research Funds of the State Key Laboratory of Ophthalmology (Number 2026QNJS04).

## Consent

The patient provided oral and written consent.

## Conflicts of Interest

The authors declare no conflicts of interest.

## Data Availability

The authors have nothing to report.
